# Quantitative Analysis of Temporal Parameters Correlated with Aspiration and Lesion Location in Stroke Patients

**DOI:** 10.1007/s00455-023-10575-0

**Published:** 2023-04-18

**Authors:** Jeong Min Kim, Ji Eun Park, Seung Jun Baek, Seung Nam Yang

**Affiliations:** 1 Department of Physical Medicine and Rehabilitation, Korea University Guro Hospital, Korea University College of Medicine, 148, Gurodong-ro, Guro-gu, Seoul, 08308 Korea; 2 Department of Computer Science and Engineering, Korea University, Seoul, 02841 Korea

**Keywords:** Dysphagia, Stroke, Videofluoroscopic swallowing study, Videofluorography, Temporal analysis

## Abstract

The purpose of this study was to identify differences in temporal parameters correlating to the presence of aspiration and the severity of penetration-aspiration scale (PAS) in patients with dysphagia after stroke. We also investigated whether there was a significant difference in temporal parameters based on the location of the stroke lesion. A total of 91 patient videofluoroscopic swallowing study (VFSS) videos of stroke patients with dysphagia were retrospectively analyzed. Various temporal parameters including oral phase duration, pharyngeal delay time, pharyngeal response time, pharyngeal transit time, laryngeal vestibule closure reaction time, laryngeal vestibule closure duration, upper esophageal sphincter opening duration and upper esophageal sphincter reaction time were measured. Subjects were grouped by the presence of aspiration, PAS score, and location of the stroke lesion. Pharyngeal response time, laryngeal vestibule closure duration, and upper esophageal sphincter opening duration were significantly prolonged in the aspiration group. These three factors showed positive correlation with PAS. In terms of stroke lesion, oral phase duration was significantly prolonged in the supratentorial lesion group, while upper esophageal sphincter opening duration was significantly prolonged in the infratentorial lesion group. We have demonstrated that quantitative temporal analysis of VFSS can be a clinically valuable tool identifying dysphagia pattern associated with stroke lesion or aspiration risk.

## Introduction

Dysphagia, difficulty of swallowing, is common in acute stroke patients. It has been reported to affect from 8.1 to 80% of post-stroke patients [1, 2]. This large variance is due to differences in the definition of dysphagia, the method of assessing swallowing function, and the timing of swallowing assessment after stroke. As dysphagia can lead to serious medical conditions including malnutrition, dehydration, aspiration pneumonia, and choking, it is important to accurately recognize and diagnose dysphagia after stroke [2–4].

Several diagnostic methods such as bedside swallowing tests, videofluoroscopic swallowing studies (VFSS), and fiberoptic endoscopic evaluation of swallowing (FEES) have been used for detection for dysphagia after stroke. VFSS and FEES both provide structural and functional information of the swallowing. However, VFSS is more useful examining entire swallowing process, because FEES has a limitation in observing pharyngeal phase problem due to the occurrence of a white-out period during some part of the pharyngeal phase. So VFSS has been considered as a gold standard examination [5–7]. Although it is a widely used assessment, reports of its validity and reliability of measurement are varied [8, 9]. To achieve a more objective and quantitative analysis for VFSS, event time of bolus and structural displacement during swallowing are analyzed for both clinical and research purposes [10–14]. Many temporal analyses of swallowing events using VFSS have been performed on normal adults [10, 11, 13, 15–21]. Although there were some studies analyzing temporal parameters in VFSS of stroke patients, most focused on a few specific parameters, and the definition of temporal parameters is often inconsistent from study to study [15, 22–34]. Therefore, consistent and detailed data on temporal parameters, which are considered clinically valuable, are needed for reliable quantitative analysis of VFSS.

The purpose of this study was to identify differences in temporal parameters depending on the presence of aspiration and the severity of penetration-aspiration scale (PAS) in stroke patients. Also, we investigated whether there was a significant difference in temporal parameters by stroke lesion location.

## Methods

### Participants

Post-stroke patients who underwent VFSS at Korea University Guro Hospital from September 2020 to October 2021 were consecutively recruited for this study. VFSS was performed in the following cases: (1) when the patient subjectively complained of dysphagia, (2) patients who were hospitalized in another department and were referred to our department for VFSS, and (3) when aspiration is suspected in the bedside water swallowing test. The water-swallowing test was performed using 50 mL of water in 10 mL aliquots. When coughing, choking, change of voice quality or oxygen desaturation ≥ 2% occurred, it was considered as suspected aspiration [35, 36]. In total, 156 VFSS videos were reviewed retrospectively.

Exclusion criteria were as follows: (1) patients with multiple stroke history, not the first onset of stroke, (2) patients younger than 19 years old, (3) patients with accompanying diseases other than stroke that could cause dysphagia, (4) patients in whom the localization of the stroke lesion was difficult such as subarachnoid hemorrhage, (5) patients unable to progress from the pharyngeal phase to the esophageal phase, such as severe upper esophageal sphincter (UES) dysfunction whose UES cannot be opened appropriately, because fully analyzing temporal parameters included in pharyngeal and esophageal phases was not possible (e.g., pharyngeal transit time, UES opening duration, etc.), (6) when there was an incompletely recorded video, and (7) when the contrast of the video was too low to identify anatomic structures.

We classified the location of stroke lesions into three groups: supratentorial, infratentorial, and both supra- and infratentorial lesions. The location was confirmed by reviewing brain images (CT or MRI) or medical records. Demographic data, time after stroke onset, and the type of stroke (ischemic or hemorrhagic) were also obtained from the medical records. This study was approved by our Institutional Research Ethics Committee for Human Subjects (IRB No. 2021GR0568).

### Videofluoroscopy

VFSS was performed by one rehabilitation physician (JM Kim) including giving bolus trials and imaging. Subjects were seated in an upright position. The lateral view of the head and neck area was recorded at frequency of 15 frames per second (FPS). VFSS was conducted using the radio-fluoroscopy system Sonialvision G4 (Shimadzu Medical Systems & Equipment, Japan). VFSS was performed using various amounts and viscosities of materials including thin liquid 2 cc, thin liquid 5 cc, semi-liquid 2 cc, semi-liquid 5 cc, semi-solid and solid materials. Of these, only the videos swallowing thin liquid 2 cc were analyzed because there were no barium residue present in oropharyngeal area, as thin liquid 2 cc was the first material tested in our hospital protocol. Thin liquid was mixed with barium at 35% w/v.

### VFSS Video Analysis Process

Analysis of videos was performed by two rehabilitation physicians. Two experienced rehabilitation physician (JM Kim and SN Yang) independently analyzed the VFSS video clips. The intraclass coefficient was 0.999 (*p* value < 0.001). If there were any disagreements between the two clinicians, consensus was reached through discussion.

The analysis process was carried out as follows:

1. Extracting the first subswallow video
2. PAS rating
3. Event labeling
4. Temporal parameter measurement

#### Extracting the First Subswallow Video

First, the total number of subswallows were measured. If there were multiple subswallows, only the first subswallow video was extracted. This was because the videos of the first passage of material without previous barium residue can be analyzed more accurately. We set the first frame as the starting point of the oral phase, and the last frame as the point between the swallow rest and the start of the next subswallow.

#### Penetration-Aspiration Scale (PAS) Rating

Airway invasion was rated on an eight-point PAS [37]. A higher score means more severe penetration or aspiration. Score 1 is normal. Penetration is scored from 2 to 5. Aspiration is scored as 6, 7, or 8. The detailed PAS classification is as follows.

1. = Material does not enter the airway.
2. = Material enters the airway, remains above the vocal folds, and is ejected from the airway.
3. = Material enters the airway, remains above the vocal folds, and is not ejected from the airway.
4. = Material enters the airway, contacts the vocal folds, and is ejected from the airway.
5. = Material enters the airway, contacts the vocal folds, and is not ejected from the airway.
6. = Material enters the airway, passes below the vocal folds, and is ejected into the larynx or out of the airway.
7. = Material enters the airway, passes below the vocal folds, and is not ejected from the trachea despite effort.
8. = Material enters the airway and passes below the vocal folds, and no effort is made to eject

#### Major Event Labeling

Major event labeling was performed similar to the *ASPEKT method* devised by Steele et al. [10]. The adjusted definitions of the major events we used are as follows:

A. Start of oral phase (SO): This event is determined as the time when the bolus first enters the oral cavity. This event is also the first frame of the video clip used for analysis.
B. Bolus past mandible (BPM): This is the first frame where the leading edge of the bolus touches or crosses the shadow of the ramus of mandible. If the two lines of the mandible do not overlap, the midpoint between the upper and lower mandible lines is taken as the reference point.
C. Burst of hyoid bone (HYB): This is the first frame when the hyoid bone starts an anterior–superior jump.
D. Laryngeal vestibule closure (LVC): This event is the first frame showing contact between the inferior surface of the epiglottis and the arytenoid process. When LVC occurs completely, air space of the laryngeal vestibule is invisible. When LVC occurs incompletely, the frame of maximum approximation of the arytenoid process to the inferior surface of the epiglottis is used.
E. Upper esophageal sphincter opening (UESO): This is the first frame when upper esophageal sphincter (UES) opens as the bolus or air passes through it.
F. Maximal pharyngeal constriction (MPC): This is the earliest frame when the space in the pharynx became minimized.
G. UES closure (UESC): This is the first frame when a single point of the UES segment closes behind the bolus tail.
H. LVC offset: This is the earliest frame when the air space of the laryngeal vestibule become visible.
I. Swallow rest: This is the last event of the swallow, when the pyriform sinuses are at their lowest position before the subsequent subswallow starts.

#### Temporal Parameter Measurement

The temporal parameters measured were as follows:

A. Oral phase duration
  Start point: Start of oral phase (SO)
  Terminal point: Bolus past mandible (BPM)
B. Pharyngeal delay time
  Start point: Bolus past mandible (BPM)
  Terminal point: Burst of hyoid bone (HYB)
C. Pharyngeal response time
  Start point: Burst of hyoid bone (HYB)
  Terminal point: upper esophageal sphincter closure (UESC)
D. Pharyngeal transit time
  Start point: Bolus point mandible (BPM)
  Terminal point: Upper esophageal closure (UESC)
E. LVC reaction time
  Start point: Burst of hyoid bone (HYB)
  Terminal point: Laryngeal vestibule closure (LVC)
F. LVC duration
  Start point: Laryngeal vestibule closure (LVC)
  Terminal point: LVC offset
G. UES opening duration
  Start point: Upper esophageal sphincter opening (UESO)
  Terminal point: Upper esophageal sphincter closure (UESC)
H. UES reaction time
  Start point: Burst of hyoid bone (HYB)
  Terminal point: Upper esophageal sphincter opening (UESO)

Since fluoroscopy was projected at 15 FPS using the frame number of the main events, the above temporal parameters could be calculated in milliseconds.

### Statistical Analysis

All analyses were conducted using IBM SPSS statistics version 26, and *p* values less than 0.05 were considered statistically significant. Normality of data was assessed using Kolmogorov–Smirnov and Shapiro–Wilk tests. The Mann–Whitney *U* test was used to compare temporal parameters between aspiration group and non-aspiration group. Then, the simple and multiple logistic regression analyses were performed to evaluate the correlation of the temporal parameters with the presence of aspiration. The Spearman correlation was utilized to evaluate the correlation temporal parameters with the PAS score. The Kruskal–Wallis test was used to compare temporal parameters between the location of stroke lesion. Quantitative data, such as temporal parameters of each patient groups, are presented as median with interquartile range. Qualitative data are reported as frequency and percentage.

## Results

### General Characteristics of the Subjects

A total of 91 from 156 patient VFSS videos were analyzed for this study. Of these, 65 videos were excluded according to the exclusion criteria. 52 (57.1%) patients were male, and 39 (42.9%) were female. The mean age was 71.6 ± 13.1 years. The time after stroke onset and the type of the stroke are also described in Table 1.

**Table 1:** General characteristics of subjects

	*n* = 91 (100%)
Age (years, mean ± SD)	71.6 ± 13.1
Gender	
Male	52 (57.1%)
Female	39 (42.9%)
Time after stroke onset (months) (median, IQR)	2.5 (0.7–11.0)
Stroke type	
Ischemic	76 (83.5%)
Hemorrhagic	15 (16.5%)
Location of stroke lesion	
Supratentorial lesion	69 (75.8%)
Cerebral cortex	5 (5.5%)
Subcortex	24 (26.4%)
Both cerebral cortical and subcortical area	40 (44.0%)
Infratentorial lesion	13 (14.3%)
Cerebellum	2 (2.2%)
Brainstem	10 (11.0%)
Both cerebellum and brainstem	1 (1.1%)
Both supra- and infratentorial lesion	9 (9.9%)
PAS	
1	64(70.3%)
2	5 (5.5%)
3	12 (13.2%)
4	0 (0.0%)
5	3 (3.3%)
6	1 (1.1%)
7	0 (0.0%)
8	6 (6.6%)
Subswallow number (mean ± SD)	1.3 ± 0.8

*SD* standard deviation, *IQR* interquartile range *PAS* penetration-aspiration scale

When classified according to the location of the stroke lesion, 69 (75.8%) patients had supratentorial lesions, 13 (14.3%) had infratentorial lesions, and 9 (9.9%) had both supra- and infratentorial lesions. The more detail data are presented in Table 1.

The PAS of subjects is described in Table 1. According to the PAS, subjects were classified into two groups. The ‘aspiration group’ included patients with PAS scores of 6 to 8. The ‘non-aspiration group’ was consisted of patients with PAS scores of 1 to 5. The number of patients classified into the ‘aspiration group’ and ‘non-aspiration group’ was 7 and 84, respectively.

### Comparison of Temporal Parameters Between Aspiration and Non-aspiration Groups

When comparing the temporal parameters of the aspiration group and the non-aspiration group, three parameters had significant differences: Pharyngeal response time (PRT), LVC duration, and UES opening duration (Table 2). All three parameters were significantly prolonged in the aspiration group.

**Table 2:** Comparison of temporal parameters between aspiration and non-aspiration groups

	Aspiration group (PAS 6 to 8) (*n* = 7)	Non-aspiration group (PAS 1 to 5) (*n* = 84)	*p* value
Oral phase duration (from ‘SO’ to ‘BPM’)	1.00 (0.27–2.00)	1.13 (0.62–2.50)	0.293
Pharyngeal delay time (PDT) (from ‘BPM’ to ‘HYB’)	0.80 (0.60–2.53)	0.77 (0.15–1.88)	0.336
Pharyngeal response time (PRT) (from ‘HYB’ to ‘UESC’)	0.73 (0.67–0.80)	0.60 (0.53–0.67)	0.009*
Pharyngeal transit time (PTT) (from ‘BPM’ to ‘UESC’)	1.53 (1.33–3.27)	1.33 (0.80–2.52)	0.200
LVC reaction time (from ‘HYB’ to ‘LVC’)	0.13 (0.13–0.33)	0.20 (0.13–0.27)	0.490
LVC duration (from ‘LVC’ to ‘LVC offset’)	1.07 (0.87–1.20)	0.60 (0.47–0.73)	0.003*
UES opening duration (from ‘UESO’ to ‘UESC’)	0.53 (0.40–0.53)	0.33 (0.27–0.47)	0.031*
UES reaction time (from ‘HYB’ to ‘UESO’)	0.20 (0.20–0.27)	0.27 (0.20–0.27)	0.441

Values are presented as median (interquartile range)

*SO* start of oral phase, *BPM* bolus past mandible, *HYB* burst of hyoid bone, *UESC* upper esophageal sphincter closure, *LVC* laryngeal vestibule closure, *LVC offset* laryngeal vestibule closure offset, *UESO* UES opening

*Significant at *p* < 0.05, by Mann–Whitney *U* test

As described at Table 3, simple and multiple logistic regression analyses were utilized to evaluate factors affecting aspiration. In simple logistic regression analysis, PRT, LVC duration, and UES opening duration were significant factors. Multiple logistic regression analysis was performed with these three factors. LVC duration was identified as the most relevant factor, which was statistically borderline significant (*p* = 0.056), affecting aspiration among these factors.

**Table 3:** Simple and multiple logistic regression analysis—risk factors for aspiration

	Simple logistic regression analysis			Multiple logistic regression analysis		
	OR	95% CI	*p* value	OR	95% CI	*p* value
Age	1.026	0.959–1.098	0.458			
Gender						
Male	0.272	0.050–1.484	0.133			
Female	3.676	0.674–20.059				
Temporal parameters						
Oral phase duration (from ‘SO’ to ‘BPM’)	0.764	0.411–1.420	0.395			
Pharyngeal delay time (PDT) (from ‘BPM’ to ‘HYB’)	0.955	0.678–1.346	0.793			
Pharyngeal response time (PRT) (from ‘HYB’ to ‘UESC’)	1059.112	1.895–591,985.495	0.031*	76.964	0.007–888,409.141	0.363
Pharyngeal transit time (PTT) (from ‘BPM’ to ‘UESC’)	0.975	0.713–1.334	0.876			
LVC reaction time (from ‘HYB’ to ‘LVC’)	0.353	0.000–1244.158	0.803			
LVC duration (from ‘LVC’ to ‘LVC offset’)	6.6673	1.320–33.732	0.022*	5.679	0.960–33.604	0.056
UES opening duration (from ‘UESO’ to ‘UESC’)	2032.147	1.165–3,543,551.249	0.045*	39.676	0.003–471,409.682	0.442
UES reaction time (from ‘HYB’ to ‘UESO’)	3.206	0.001–7568.057	0.769			

*SO* start of oral phase, *BPM* bolus past mandible, *HYB* burst of hyoid bone, *UESC* upper esophageal sphincter closure, *LVC* laryngeal vestibule closure, *LVC offset* laryngeal vestibule closure offset, *UESO* UES opening

*Significant at *p* < 0.05

### Correlation Between Temporal Parameters and PAS

To understand the correlation between temporal parameters and PAS severity, we used Spearman’s correlation coefficients. PRT (*R* = 0.220, *p* value = 0.036), LVC duration (*R* = 0.268, *p* value = 0.010) and UES opening duration (R = 0.230, *p* value = 0.028) were significantly correlated with the PAS score.

### Comparison of Temporal Parameters by Location of Stroke Lesion

As mentioned in Table 4, oral phase duration and UES opening duration were two parameters that differed significantly by the location of stroke lesion. Oral phase duration was significantly prolonged in the ‘supratentorial lesion group’ and ‘both supra- and infratentorial group’ compared with the ‘infratentorial lesion group’. For UES opening duration, significant differences were identified between all the three groups. UES opening duration was longest at the infratentorial lesion group.

**Table 4:** Comparison of temporal parameters by location of stroke lesion

	Supratentorial lesion (*n* = 69)	Infratentorial lesion (*n* = 13)	Supra- and infratentorial lesion (*n* = 9)	*p* value
Oral phase duration (from ‘SO’ to ‘BPM’)	1.33 (0.63–2.70)	0.67 (0.43–0.97)	2.40 (0.57–5.67)	0.024*
Pharyngeal delay time (PDT) (from ‘BPM’ to ‘HYB’)	0.80 (0.20–2.07)	0.47 (0.20–0.27)	0.60 (0.13–2.37)	0.983
Pharyngeal response time (PRT) (from ‘HYB’ to ‘UESC’)	0.60 (0.53–0.73)	0.67 (0.57–0.80)	0.60 (0.50–0.63)	0.197
Pharyngeal transit time (PTT) (from ‘BPM’ to ‘UESC’)	1.40 (0.80–2.70)	1.13 (0.90–3.33)	1.27 (0.70–2.93)	0.964
LVC reaction time (from ‘HYB’ to ‘LVC’)	0.20 (0.13–0.27)	0.20 (0.13–0.27)	0.20 (0.13–0.27)	0.646
LVC duration (from ‘LVC’ to ‘LVC offset’)	0.60 (0.47–0.73)	0.67 (0.47–0.83)	0.87 (0.50–0.97)	0.376
UES opening duration (from ‘UESO’ to ‘UESC’)	0.33 (0.27–0.47)	0.47 (0.37–0.53)	0.27 (0.20–0.33)	0.004*
UES reaction time (from ‘HYB’ to ‘UESO’)	0.27 (0.20–0.27)	0.20 (0.13–0.30)	0.27 (0.27–0.37)	0.110

Values are presented as median (interquartile range)

*SO* start of oral phase, *BPM* bolus past mandible, *HYB* burst of hyoid bone, *UESC* upper esophageal sphincter closure, *LVC* laryngeal vestibule closure, *LVC offset* laryngeal vestibule closure offset, *UESO* UES opening

*Significant at *p* < 0.05, by Kruskal–Wallis test

## Discussion

We demonstrated that prolonged PRT, LVC duration and UES opening duration were negatively correlated with swallowing function with stroke patients indicating significantly increased risk of aspiration. In addition, oral phase duration and UES opening duration were significantly prolonged in supratentorial and infratentorial lesion of stroke, respectively.

Many previous temporal analytical studies focused on pharyngeal delay time (PDT) or pharyngeal transit time (PTT) [17, 25–31, 38–42], whereas few studies focused on pharyngeal response time (PRT). The term PRT has been used interchangeably with PDT [41] or defined as a completely different time interval [17, 38–40]. In our study, we used PDT and PRT separately as defined by Logemann et al. [17, 39, 40]. Previous studies suggested that prolonged PDT is associated with aspiration [18, 26–28, 42]. Prolonged PTT was also reported to increase the risk of aspiration [25, 29–31]. However, in our study, there were no significant differences in PDT or PTT between the aspirators and non-aspirators, but PRT was significantly longer in the aspirator group compared with the non-aspirator group (Tables 2, 3). To the best of our knowledge, importance of PRT was first demonstrated in our study. In addition to PDT and PTT, which were suggested as important parameters in the pharyngeal phase in the previous studies, it would be helpful to analyze dysphagia in detail if PRT is included in temporal analysis.

When contrast enters laryngeal vestibule, this is called penetration, which can lead to aspiration that enters beyond the vocal cord. Therefore, it is important that laryngeal vestibule closure (LVC) occurs properly in an appropriate timing in the pharyngeal phase to prevent penetration or aspiration [32, 43–45]. Two temporal parameters were analyzed for the laryngeal vestibule closure: LVC reaction time (how quickly the laryngeal vestibule get closed at the beginning of the pharyngeal phase) and LVC duration (how long the laryngeal vestibule remains closed). Regarding the LVC reaction time, no significant differences were observed in our study depending on the presence of aspiration. There were some studies in which LVC reaction time was significantly prolonged in aspirators. Kahrilas et al. [44] suggested that prolonged LVC reaction time and UES reaction time were key abnormalities leading to laryngeal penetration or aspiration. Cabib et al. [32] also suggested that prolongation of total oropharyngeal swallow response along with prolonged LVC reaction time and UES opening reaction time were seen in stroke aspirators. Park et al. [23] reported that initiation of laryngeal closure was significantly prolonged in the stroke aspirators compared with stroke non-aspirators or normal subjects. In these previous studies, the starting point of the LVC reaction time was set as BPM or glossopalatal junction opening, whereas in our study, it was set as HYB. However, if the starting point of the LVC reaction time is set to the same point as in previous studies, it is difficult to purely measure the time it takes for the laryngeal vestibule to close because the PDT is included in the LVC reaction time. Therefore, we set the starting point of the LVC reaction time as HYB, based on the study of Steele et al. [10, 16]; our settings have led to different results from the previous studies.

In this study, prolonged LVC duration was significantly associated with the presence of aspiration or PAS severity. Prolonged LVC duration may be caused by prolonged swallowing time. In our results, aspirators showed prolonged pharyngeal phase (PTT), although this was not statistically significant, as well as significantly prolonged LVC duration compared to non-aspirators. However, this result was not consistent with previous studies. Power et al. [27] reported that there was no significant difference in LVC duration between normal subjects, stroke non-aspirators, and stroke aspirators. Park et al. [23] reported that LVC duration was significantly shorter in stroke aspirators compared with normal subjects. The inconsistency of the results of these studies, including ours, indicates that it is difficult to predict aspiration risk simply by the length of the LVC duration. Rather, appropriate timing and length of laryngeal vestibule closure are important parameters to investigate regarding aspiration prevention.

The UES should be opened at the appropriate timing for efficient and safe swallowing. If the UES does not open appropriately, bolus transition from pharynx to esophagus become difficult, which can lead to a large amount of pharyngeal remnant and result in aspiration. Some previous studies have discussed the relationship between UES opening duration and aspiration. Kim et al. [33] found that prolonged UES opening duration is related to aspiration, which results in retrograde flow increasing aspiration risk. They suggested that UES stays open longer due to prolonged bolus transition, slower UES muscle contraction and hyolaryngeal excursion after stroke. Consistently, in our study, prolonged UES opening duration was associated with aspiration and higher PAS score. In contrast, Molfenter and Steele [24] suggested that in stroke patients, aspirators demonstrated significantly shorter UES opening duration compared with non-aspirators. The reason their results are not consistent with ours is likely because they set the definition of their aspiration group to PAS 3 or higher, which is different from the commonly used criteria. Therefore, the results of our study, which set the criteria of aspiration as PAS 6 or higher, can be considered more clinically meaningful results.

Although there were some studies comparing the pattern of dysphagia between supratentorial and infratentorial strokes, few studies quantitatively compared temporal parameters of VFSS. Daniels et al. [46] identified that a higher PAS score is more directly associated with infratentorial stroke than supratentorial stroke, but they did not analyze the temporal parameter. A previous study by Kim et al. [34] reported that the pharyngeal remnant and PAS score were more severe in infratentorial stroke patients than in supratentorial stroke patients. However, they suggested that there were no significant differences in the PTT and functional dysphagia scale. Similarly, in our study, there was no significant difference in the PTT by stroke lesion location.

Previous studies have reported that the oral phase of swallowing is affected by cognitive function [47–49]. Jo et al. [48] reported that cognitive function had a meaningful influence on the oral phase in supratentorial stroke patients. Moon et al. [47] found that the coordination of the lips, tongue, and oropharynx was associated with the degree of cognitive impairment and stroke severity. Consistent with these results, in our study, we found that oral phase duration was significantly prolonged in supratentorial stroke patients compared with infratentorial stroke patients.

Similarly, in our study, UES opening duration was significantly prolonged in the infratentorial lesion group compared to the supratentorial group. Lower brainstem is important for swallow response in pharyngeal phase as central pattern generator and adjacent medullary reticular formation are situated. Therefore, infratentorial stroke, such as lateral medullary syndrome, can result in UES dysfunction [32, 50–52]. Steinhagen et al. [50] suggested that brainstem stroke patients may experience UES and pharyngeal incoordination, UES abnormal relaxation, and closure. Another study suggested that up to 80% of patients of brainstem stroke have dysphagia by failed UES relaxation [51]. However, in the present study, patients with severe UES dysfunction were excluded due to difficulty in temporal analysis, so the results may be less accurate. Future studies that include these patients may demonstrate different results.

This study has some limitations. We used fluoroscopy machine of 15 FPS unlike many previous studies that used 30 FPS machine. Although this allowed us to reduce the radiation dose for the patients, the results of temporal analysis could have been less accurate. Since this was a retrospective and cross-sectional study, this may have resulted in imbalance in the number of samples between groups, inconsistency in the time after stroke onset and the distribution of dysphagia severity. In addition, we did not have any control group to compare the results of stroke patients. Therefore, a prospective study with a large number of balanced samples between groups, including the control group, should be performed in the future. Moreover, subjects who could not progress from the pharyngeal phase to the esophageal phase due to severe UES dysfunction were excluded from this study because fully measuring temporal parameters included in pharyngeal and esophageal phases was impossible in these cases. Because many of these patients excluded had infratentorial lesions, a sufficient number of infratentorial lesion group subjects could not be recruited. In addition, only the videos swallowing thin liquid 2 cc were used for analysis because there was no previous barium residue present since in our hospital protocol, thin liquid 2 cc is tested first. Similarly, only the videos of first subswallow were included in this study, because it was easy to accurately analyze the videos in which existing barium residue does not remain in the oropharyngeal area. However, as many post-stroke patients have multiple subswallows per bolus and more severe penetration or aspiration could occur on additional subswallows. Therefore, this may have resulted in underestimation of the severity of penetration or aspiration. If further study including not only various volumes and viscosities of materials but also analyzing multiple subswallows, more accurate and clinically applicable results could be obtained.

## Conclusions

This study found that temporal analysis of VFSS can be a clinically valuable tool to help understand the dysphagia patterns related to aspiration and location of brain lesions in stroke patients. More accurate comparative analyses will be possible if subjects are recruited prospectively and a control/non-stroke group is included. Follow-up exams will also need to be performed in future studies.
